# The effect of coffee thermocycling and color correction serum on the colorimetric properties and hardness of CAD‐CAM restorative materials

**DOI:** 10.1111/jopr.70089

**Published:** 2026-01-09

**Authors:** Hanan Al‐Johani

**Affiliations:** ^1^ Faculty of Dentistry, Department of Restorative Dentistry King Abdulaziz University Jeddah Saudi Arabia

## Abstract

**Purpose:**

To compare the stainability, translucency, opalescence, whiteness, gloss, and hardness of 4 computer‐aided design and computer‐aided manufacturing (CAD‐CAM) restorative materials after simulated coffee drinking and color correction.

**Materials and Methods:**

Four CAD‐CAM blocks were investigated (n = 40): resin nanoceramic (Cerasmart, CS), polymer‐infiltrated ceramic network (Vita Enamic, VE), lithium disilicate glass ceramic (IPS e.max CAD, EC), and zirconia‐reinforced lithium silicate glass ceramic (Vita Suprinity, VS). The color stability (Δ*E*_00_), relative translucency (RTP), opalescence (OP), and whiteness stability (ΔWI_D_) were measured with a spectrophotometer. Gloss (GU) was recorded with a gloss meter, and hardness (HM) was detected with a Martens hardness testing device. The specimens were observed at baseline (T0), after coffee thermocycling (CTC) (T1), and after color correction (T2). Δ*E*_00_ was analyzed by two‐way ANOVA, ΔWI_D_ was analyzed with one‐way ANOVA, and RTP, OP, WI_D_, GU, and HM were assessed with repeated ANOVA to evaluate the effects of material type, treatment, and their interactions on the tested properties. Post hoc pairwise comparisons were assessed by Tukey HSD and Student *t*‐tests (*α* = 0.05).

**Results:**

Δ*E*_00_, WI_D_, OP, GU, and HM outcomes were significantly impacted by material type (*p* < 0.001) and treatment (*p* ≤ 0.028), whereas ΔWI_D_ and RTP were affected by material type (*p* ≤ 0.03). CS exceeded the Δ*E*_00_ perceptibility threshold at T1. VS exhibited the highest stain resistance, RTP, and HM, and the least was in VE and CS. Color correction serums reversed CTC‐induced staining among VS and VE (*p* < 0.001).

**Conclusions:**

Zirconia‐reinforced lithium silicate glass‐ceramics demonstrated superior color stability and hardness when subjected to coffee thermocycling and color correction serum.

Advancements in computer‐aided design and computer‐aided manufacturing (CAD‐CAM) technologies have facilitated efficient manufacturing of monolithic dental restorations from restorative materials in an array of compositions.^1^ The longevity of monolithic CAD‐CAM restorations is contingent on their ability to fulfill high esthetic demands and deliver optimum mechanical performance.^2^ Traditionally, machinable glass‐ceramics have been at the forefront for prosthetic single‐unit applications by virtue of their superior esthetic and biocompatibility traits.^3^ In an attempt to enhance the mechanical durability of lithium disilicate glass‐ceramics (LD), a variety of filler reinforcements, namely zirconium dioxide (ZrO_2_) or ß‐spodumene (LiAlSi_2_O_6_), have been incorporated into the microstructure, giving rise to zirconia‐reinforced lithium silicates (ZLS) and lithium–aluminum disilicates.^4^, ^5^, ^6^ Furthermore, recently developed CAD‐CAM resin‐based materials such as the polymer‐infiltrated ceramic network (PICN) and resin nanoceramic (RNC) have also demonstrated comparable performance, proving them to be suitable alternatives to their machinable glass‐ceramic counterparts.^7^ While resin‐based materials offer advantages such as ease of milling and intraoral repair, they pose the risk of inferior wear resistance and, in turn, higher surface roughness, color change, and patient dissatisfaction.^8^, ^9^

Esthetic outcomes of monolithic dental restorations are conditional on their colorimetric properties, translucency, and stain resistance.^10^, ^11^ Additionally, inherent material properties such as chemical composition, microstructure, and filler loading considerably influence their optical behavior, surface integrity, and mechanical performance.^12^ Nevertheless, environmental intraoral dynamics may alter the appearance and sustainability of esthetic dental restorations due to temperature fluctuations and staining solutions such as coffee. Exposing dental restorative materials to coffee thermocycling (CTC) regimens induces residual stresses through thermal fluxes by means of highly concentrated colorant solutions, thus triggering physical‐chemical modifications within the surface layers and bulk material structure. The effects of CTC have been previously explored regarding LDs^13^, ^14^, ^15^, ^16^, ^17^, ^18^, ^19^ and ZLSs^13^, ^15^, ^17^, ^18^, ^19^, ^20^, ^21^; however, limited studies have investigated its impact on PICNs,^14^, ^21^, ^22^, ^23^ or RNCs.^14^, ^24^ Moreover, the existing literature evidence on CTC was conducted in relation to roughness,^21^, ^24^, ^25^, ^26^, ^27^ stainability,^13^, ^14^, ^19^, ^20^, ^21^, ^22^, ^23^, ^24^, ^27^, ^28^ translucency,^14^, ^17^, ^19^, ^20^, ^22^, ^23^, ^27^, ^28^ opalescence,^17^ Vickers hardness,^27^ fracture load,^28^ and flexural strength.^15^ The Martens hardness test uses instrumented indentation under load‐controlled settings to quantify parameters including hardness, elastic modulus, indentation depth, and creep, by virtue of objective force–displacement data, providing an accurate distinction between elastic, viscoelastic, and plastic responses.^29^ However, to date, no studies have assessed the influence of CTC on the whiteness, gloss, or Martens hardness of restorative dental materials.

Color correction serums such as the HiSmile v34 (Hismile Pty Ltd) have been recently introduced as hydrogen peroxide‐free temporary solutions to reverse the staining of discolored teeth.^30^ Therein, the radical‐free concept involves the application of violet and blue dyes to simultaneously incite the reflection of shorter visible light wavelengths from teeth, such as violet and blue, and suppress the reflection of longer wavelengths, such as yellow and brown.^31^ The effects of color correction serum were investigated in terms of natural tooth structures^30^, ^32^, ^33^; however, the inevitable proximity of dental restorations within the oral cavity heightens the demand for further exploration of their influence by color correction serums. To the best of the author's knowledge, no study has previously assessed the impact of color correction serums on CAD‐CAM restorative materials. Thereby, the null hypotheses were (1) CTC and color correction would not significantly affect the color stability, relative translucency, opalescence, whiteness stability, gloss, and hardness of CAD‐CAM restorative materials, and (2) different CAD‐CAM restorative materials would exhibit the same color stability, relative translucency, opalescence, whiteness stability, gloss, and hardness irrespective of the treatment.

## MATERIALS AND METHODS

**FIGURE 1:**
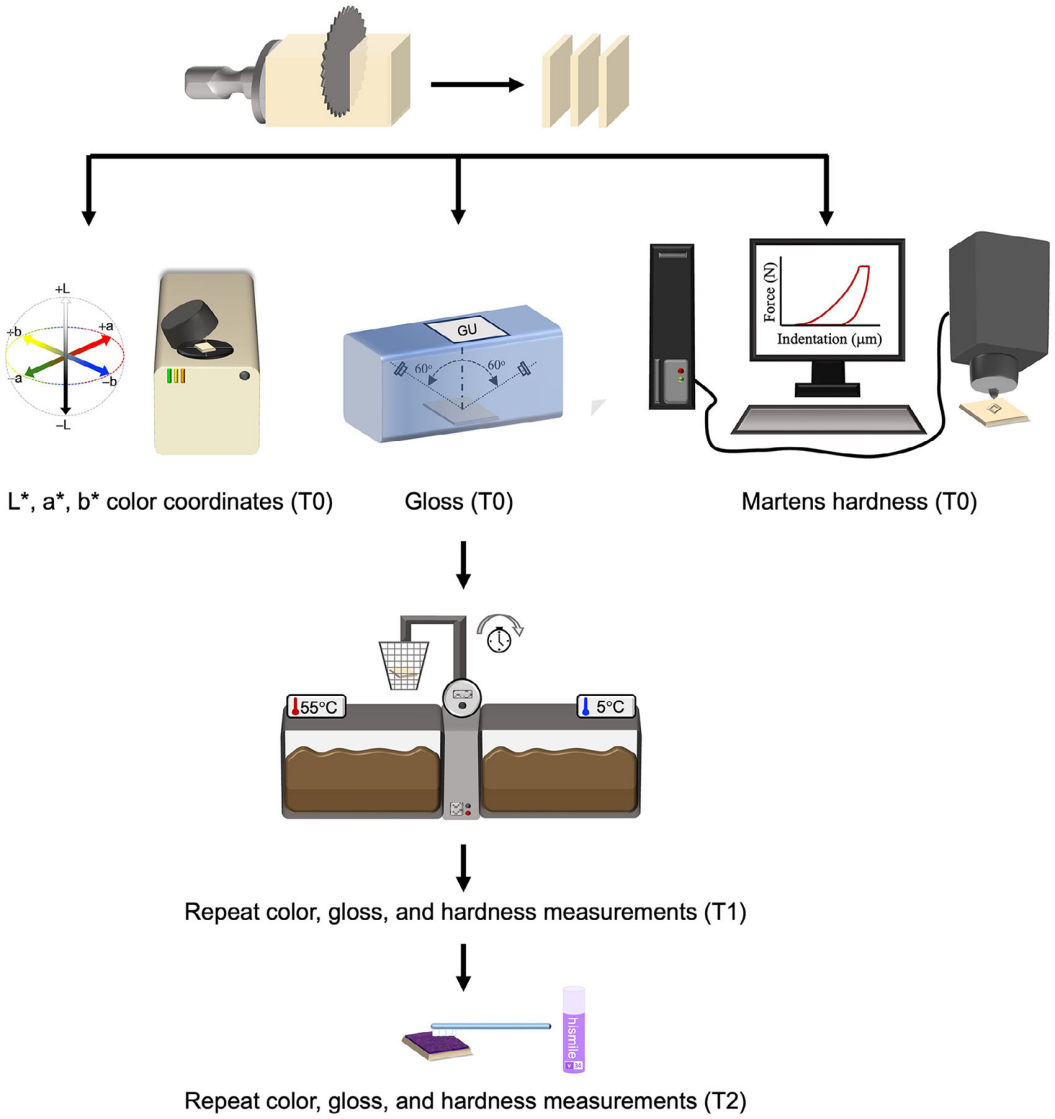
Schematic representation of experimental study design. T0, baseline; T1, after coffee thermocycling; T2, after color correction.

Figure 1 presents a summary of the study outline. Four CAD‐CAM blocks were investigated (*n* = 40): resin nanoceramic (CS: Cerasmart, GC‐Corp); polymer‐infiltrated ceramic network (VE: Vita Enamic, Vita Zahnfabrick); lithium disilicate glass ceramic (EC: IPS e.max CAD, Ivoclar Vivadent); and zirconia‐reinforced lithium silicate glass ceramic (VS: Vita Suprinity, Vita Zahnfabrik). All blocks were of similar shade (A1) and translucency level (HT) (Table 1). The sample size (*n* = 10 per material) was deemed sufficient to obtain a power of 90% based on a power analysis conducted in a similar study.^34^ The machinable blocks were sectioned into 12 × 14 × 1 mm slices using a slow‐speed diamond blade (MK 303, MK Diamond) mounted on a saw device (Isomet 1000 Precision Saw; Buehler Co.) under running water. Subsequently, both sides of the specimens were wet‐ground using a grinding device (MetaServ 250; Buehler Co.) with a sequence of silicon carbide papers (P400, P600, P1200, and P1500 grit; Buehler Co.) and polished with 0.15 µm diamond suspension (Meta Di Supreme; Buehler Co.). EC and VS specimens were crystallized in a furnace (Programat EP 5000; Ivoclar AG) according to the manufacturers' instructions. EC underwent a two‐step crystallization regime with a 403°C standby temperature, 6 min closing time, 90°C/min first heating rate, 820°C first firing temperature, and a 10 min first holding time, followed by a 30°C/min second heating rate, 840°C second firing temperature, a 7 min second holding time, and 700°C long‐term cooling. VS underwent a one‐step crystallization regime with a 400°C standby temperature, 4 min closing time, 55°C/min heating rate, 840°C firing temperature, and an 8 min‐holding time, followed by 680°C long‐term cooling. All specimens were cleaned ultrasonically in distilled water for 10 min (Ultrasonic Cleaning System; L&R Co). The outcomes of interest were measured at baseline (T0), after CTC (T1), and after color correction (T2).

**TABLE 1:** Experimental materials and manufacturers’ information.

Classification	Product name	Manufacturer	Chemical composition
Resin nanoceramics	CeraSmart	GC‐Corp	Monomer: Bis‐MEPP, UDMA, DMA Filler: SiO_2_, barium glass
Polymer‐infiltrated ceramic network	Vita Enamic	Vita Zahnfabrik	Monomer: UDMA, TEGDMA Filler: SiO_2_, Al_2_O_3_, Na_2_O, K_2_O, B_2_O_3_, ZrO_2_, CaO
Lithium disilicate glass‐ceramic	e.max CAD	Ivoclar Vivadent	SiO_2_, Li_2_O, K_2_O, P_2_O_5_, ZrO_2_, ZnO, Al_2_O_3_, MgO, pigments
Zirconia‐reinforced lithium silicate glass‐ceramic	Vita Suprinity	Vita Zahnfabrik	SiO_2_, Li_2_O, ZrO_2_, P_2_O_5_, Al_2_O_3_, K_2_O, CeO_2_, La_2_O_3_, pigments
Whitening gel	HiSmile	HiSmile Pty Ltd.	Glycerin, aqua/water, sorbitol, hydrated silica, xylitol, polysorbate 80, cellulose gum, Mentha piperita oil, phenoxyethanol, sucralose, tetrasodium pyrophosphate, Cl17200/D&C Red No. 33, Cl42090/FD&C Blue No. 1, ethylhexylglycerin

Martens hardness of the specimens was calculated using a hardness testing machine (Z2.5; ZwickRoell Ltd) from the following equation: $\mathrm{HM}=\frac{F}{A_{s}\left(h\right)\;}$, where HM is the Martens hardness (N/mm^2^), *F* is the maximum load (N), *As(h)* is the surface area (mm^2^) of the indenter at a distance *h* from the tip. A force‐controlled setting was employed to impose 4 indentations per specimen of 3 mm spacing, with a 10 N load, at 0.5 mm/min‐speed, and a 10‐s dwell time.^35^ The Poisson ratio (*v*) was derived from previous studies (VS, EC = 0.216, VS = 0.208, VE and CS = 0.35, and diamond indenter = 0.07.^12^, ^36^, ^37^ Mean HM values were computed from the force‐indentation curves via the equipped software program (TestXpert; Zwick GmbH Co.).^38^, ^39^

Color measurements were determined with a benchtop UV–visible light spectrophotometer (LabScan XE; Hunter Associates Laboratory Inc.) with a 5‐mm aperture that utilized the Commission International de l'Eclairage Standard (CIE), a 10° human observer characteristics, and a D65 illuminant to record the color coordinates L*, a*, and b* within a wavelength range of 400–700 nm at 10 nm intervals.^40^ Three readings of L*, a*, and b* were obtained per specimen against a black background (L* = 0.01, a* = −0.02, b* = 0.01) and against a white background (L* = 90.35, a* = −1.31, b* = −0.27),^41^ and the average L*, a*, and b* values were recorded. The relative translucency parameter (RTP) was computed with the CIEDE2000 color difference formula: $RTP=\sqrt{{\left(\frac{L_{B}-L_{W}}{K_{L}S_{L}}\right)}^{2}+{\left(\frac{C_{B}-C_{W}}{K_{C}S_{C}}\right)}^{2}+{\left(\frac{\Delta H}{K_{H}S_{H}}\right)}^{2}+R_{T}\left(\frac{\Delta C}{K_{C}S_{C}}\right)\left(\frac{\Delta H}{K_{H}S_{H}}\right)}$,^42^ and the opalescence parameter (OP) was computed by: $OP=\sqrt{{\left(a_{B}^{*}-a_{W}^{*}\right)}^{2}+{\left(b_{B}^{*}-b_{W}^{*}\right)}^{2}}$, where subscripts *B* and *W* refer to *a* ^*^ and *b* ^*^ coordinates against black and white settings.^17^, ^43^ Whiteness was reported using the whiteness index (WI_D_)^44^, ^45^ derived from *L* ^*^, *a* ^*^, and *b* ^*^ coordinates against a white background using the equation: $WI_{D}=0.511L^{*}-2.324a^{*}-1.100b^{*}$.

Gloss readings were obtained by a gloss meter (LS193; Shenzhen Linshang Technology Co) with a 60° projection angle.^46^ Specimens were positioned within a custom mold and covered with the glossmeter lid to ensure complete elimination of external light. Two gloss readings were recorded per specimen^47^ and the mean was reported in gloss units (GU), where GU = 0 is an absolute nonreflective surface and GU = 100 is an absolute refractive surface. Gloss was considered clinically acceptable when GU > 40, based on established thresholds.^48^

Subsequently, after baseline measurements, specimens were subjected to CTC of 10,000 cycles in a coffee solution (THE‐1100 thermocycler; SD Mechatronik) at 5°C and 55°C with a 30‐s dwell time and a 10‐s transfer, to simulate 1 year of intraoral use.^49^ A fresh solution was prepared and renewed every 12 h by adding 3.6 g of instant coffee powder (Nescafe Classic; Nestle) to 300 mL of hot water.^18^ After CTC, the specimens were cleaned of coffee residue by circumferential brushing 10 times with toothpaste (Crest; Procter and Gamble) and a soft‐bristle toothbrush under running water, then ultrasonically cleaned for 10 min in distilled water.^50^ Subsequently, specimens underwent 2 cycles of color correction treatment wherein 2 pumps of the color correction serum (HiSmile v34; Hismile Pty Ltd) were applied to a soft‐bristle toothbrush, and the specimens were gently brushed circumferentially for 2 min, then rinsed under running water for 1 min.^30^

L*, a*, and b* coordinates were remeasured at T1 and T2, and the color stability (Δ*E*_00_) was computed using the CIEDE2000 color difference formula^51^ with the parametric factors set as 1. Color differences were deemed as imperceptible (Δ*E*_00_ ≤ 0.8), perceptible (Δ*E*_00_ > 0.8), perceptible but clinically acceptable (0.8 < Δ*E*_00_ ≤ 1.8), moderately unacceptable (1.8 < Δ*E*_00_ ≤ 3.6), clearly unacceptable (3.6 < Δ*E*_00_ ≤5.4), or extremely unacceptable (Δ*E*_00_ > 5.4).^52^ Likewise, RTP and OP measurements were also recomputed at T1 and T2. WI_D_ measurements were repeated at T2, and ΔWI_D_ was calculated by: $\Delta WI_{D}=WI_{D2}-WI_{D0}$, where subscripts 0 and 2 refer to measurements recorded at the T0 and T2 time points. Whiteness differences were considered visually perceptible when ΔWI_D_ > 0.72 and acceptable when ΔWI_D_ < 2.62.^53^ To prevent unfavorable influence of hardness indentations on the colorimetric measurements, the color coordinates were obtained from the specimens’ side opposing the indentations.

Statistical analyses were performed by a software program (IBM SPSS Statistics, v29.0; IBM Corp). Data normality was confirmed by the Kolmogorov–Smirnov test, and homogeneity was confirmed by the Levene test. Two‐way ANOVA was employed to evaluate the effects of material type, treatment, and their interactions on the Δ*E*_00_. One‐way ANOVA was used to assess the ΔWI_D_ among materials after color correction. A repeated measures one‐way ANOVA was used to analyze RTP, OP, WI_D_, GU, and HM (between‐subject factor: material, and within‐subject factor: time). Post hoc Tukey and Student *t*‐tests were employed to identify significant pairwise comparisons within and between different materials and treatment types (*α* = 0.05).

## RESULTS

**FIGURE 2:**
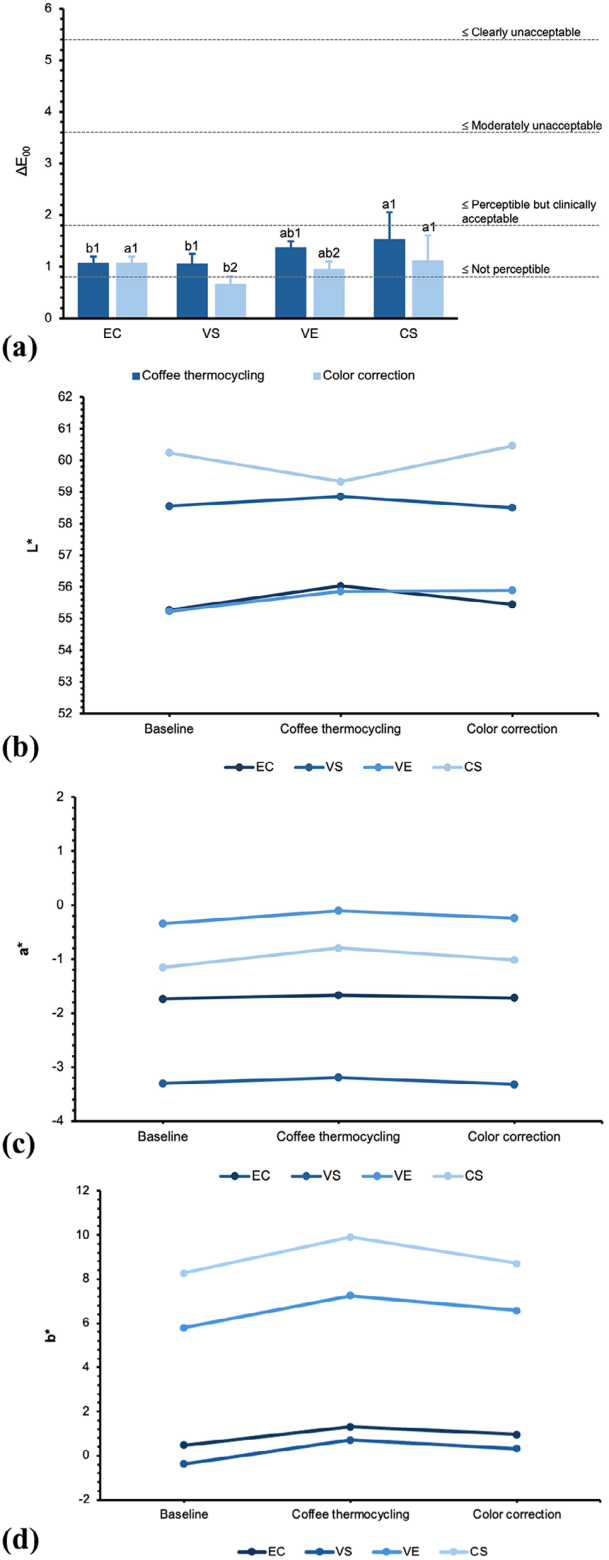
(a) Color stability (Δ*E*_00_). (b) Changes in the L* color coordinate. (c) Changes in the a* color coordinate. (d) Changes in the b* color coordinate. EC, IPS e.max CAD; VS, VITA Suprinity; VE, VITA Enamic; CS, Cerasmart. Different letters reveal significant differences among different CAD‐CAM restorative materials within the same timepoint, and different numbers indicate differences within the same CAD‐CAM restorative material across different timepoints (*p* < 0.05).

**FIGURE 3:**
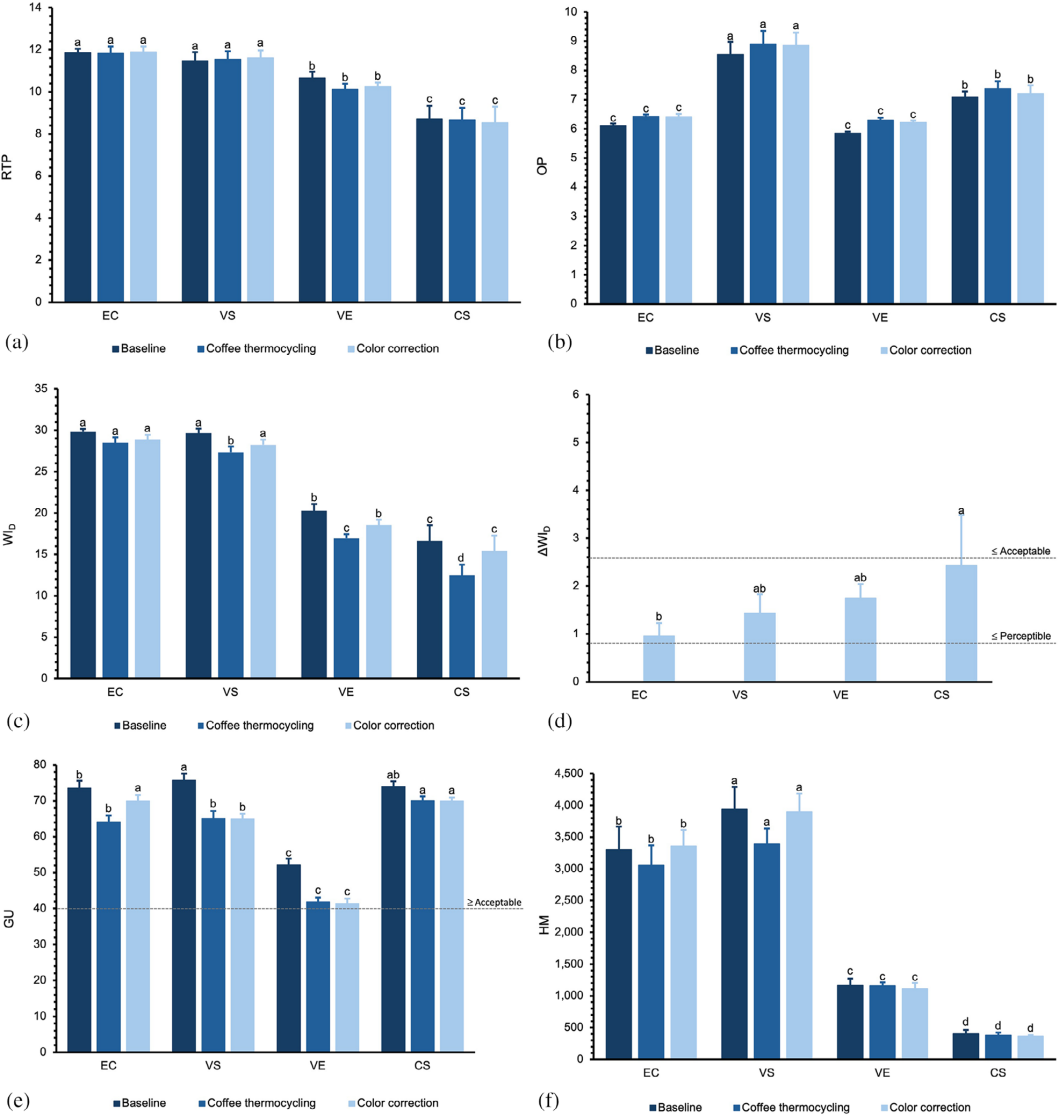
(a) Relative translucency (RTP), (b) opalescence (OP), (c) whiteness index (WI_D_), (d) whiteness stability (ΔWI_D_), (e) gloss (GU), (f) Martens hardness (HM). EC, IPS e.max CAD; VS, VITA Suprinity; VE, VITA Enamic; CS, Cerasmart; T0, baseline; T1, coffee thermocycling; T2, color correction. Different letters reveal significant differences among CAD‐CAM restorative materials within the same timepoint (*p* < 0.05).

Mean Δ*E*_00_ and changes in color coordinates L*, a*, and b* of the CAD‐CAM restorative materials after CTC and color correcting are illustrated in Figure 2 and descriptive ΔWI_D_, WI_D_, RTP, OP, GU, and HM data are presented in Figure 3. The ANOVA results of Δ*E*_00_ and ΔWI_D_ are shown in Table 2, and the repeated measures ANOVA for WI_D_, RTP, OP, GU, and HM are shown in Table 3, wherein the Mauchly test was not significant (*p* > 0.05); therefore, the assumption of sphericity had not been violated.

**TABLE 2:** ANOVA table for color stability (Δ*E*_00_) and whiteness stability (ΔWI_D_) of CAD‐CAM restorative materials subjected to coffee thermocycling and color correction.

Parameter	Source of variation	Sum of squares	df	*F*	*p*	*η*_p_^2^
Δ*E*_00_	Material	2.248	3	9.152	<0.001^*^	0.276
	Treatment	1.882	1	22.985	<0.001^*^	0.242
	Material × Treatment	0.628	3	2.556	0.062	0.096
ΔWI_D_	Material	11.351	3	3.335	0.03	0.217

Abbreviations: df, degrees of freedom (*N* − 1); *F*, *F* value; *p*, *p*‐value; *η*_p_^2^, partial eta squared.

* Statistically significant (*p* < 0.05).

**TABLE 3:** Repeated measures ANOVA table for relative translucency parameter (RTP), opalescence (OP), gloss (GU), and Martens hardness (HM) of CAD‐CAM restorative materials subjected to coffee thermocycling and color correction.

Parameter	Sum of squares	df	*F*	*p*	*η*_p_^2^
WI_D_	28.459	6	5.782	<0.001^*^	0.325
RTP	1.941	6	1.179	0.327	0.089
OP	0.229	6	2.533	0.028^*^	0.174
GU	353.281	6	24.55	<0.001^*^	0.672
HM	1,385,651.69	6	4.526	<0.001^*^	0.274

Abbreviations: df, degrees of freedom (*N* − 1); *F*, *F* value; *p*, *p*‐value; *η*_p_^2^, partial eta squared.

* Statistically significant (*p* < 0.05). Between‐subject factor: material, within‐subject factor: time.

The ΔE_00_ was significantly impacted by both main factors, material (*p* < 0.001, *η*_p_^2^ = 0.276) and treatment (*p* < 0.001, *η*_p_^2^ = 0.242), while the interaction between both factors was not significant (*p* = 0.062). CS exceeded the perceptibility threshold at T1, whereas no materials surpassed the Δ*E*_00_ acceptability threshold, regardless of the treatment type. CS displayed significantly higher Δ*E*_00_ than EC and VS at T1 (*p* ≤ 0.005), and CS and EC had significantly higher Δ*E*_00_ than VS at T2 (*p* ≤ 0.01). VS and VE exhibited lower Δ*E*_00_ at T2, whereas EC and CS displayed similar Δ*E*_00_ after both treatments (*p* ≥ 0.087). Regardless of treatment type, CS displayed the highest L* and b* coordinates, whereas VS had the lowest a* and b*.

RTP was considerably impacted by material (*p* < 0.001, *η*_p_^2^ = 0.951), whereas the impact of treatment was not significant (*p* = 0.327, *η*_p_^2^ = 0.089). Significantly higher RTP was observed in EC and VS, followed by VE, and lowest in CS (*p* < 0.001) across all treatment types. OP was impacted by material (*p* < 0.001, *η*_p_^2^ = 0.957) and treatment (*p* = 0.028, *η*_p_^2^ = 0.174). VS had significantly greater OP than other materials, followed by CS (*p* < 0.001), whereas EC and VE demonstrated similar OP throughout all treatments (*p* ≥ 0.109). WI_D_ was significantly influenced by material (*p* < 0.001, *η*_p_^2^ = 0.989) and treatment (*p* < 0.001, *η*_p_^2^ = 0.325). At T0 and T2, EC and VS demonstrated higher WI_D_ than VE and CS (*p* < 0.001). After CTC, EC showed significantly greater WI_D_, followed by VS, with the lowest values observed in CS (*p* < 0.001). VS, VE, and CS exhibited significantly higher WI_D_ at T2 than at T1. The ΔWI_D_ was impacted by material (*p* < 0.03, *η*_p_^2^ = 0.217); all materials surpassed the ΔWI_D_ perceptibility limit, and CS exceeded the ΔWI_D_ acceptability limit. GU was affected by material (*p* < 0.001, *η*_p_^2^ = 0.993) and treatment (*p* < 0.001, *η*_p_^2^ = 0.672). Regardless of the treatment type, all materials exhibited acceptable gloss values when previous thresholds were considered.

HM was impacted by material (*p* < 0.001, *η*_p_^2^ = 0.274) and treatment type (*p* < 0.001, *η*_p_^2^ = 0.672). Irrespective of treatment type, the highest HM was exhibited by VS, followed by EC, and the least was detected in CS (*p* < 0.001).

## DISCUSSION

CTC and color correction significantly altered the colorimetric properties and hardness of CAD‐CAM restorative materials. Moreover, irrespective of the treatment employed, different CAD‐CAM restorative materials displayed dissimilar colorimetric characteristics and hardness. Thus, both null hypotheses of the present study were rejected.

The novelty in this scientific body lies in its comprehensive characterization of chemically diverse CAD‐CAM restorative materials. Moreover, the implementation of CTC further enhances the clinical relevance of the study, as it provides an accurate simulation of coffee drinking among patients during a 1‐year period. Additionally, while previous studies have explored staining and color stability, the inclusion of a post‐staining color correction intervention herein, presents a practical clinical interpretation. Specimens were standardized in terms of shade, translucency, thickness, and polishing protocol. Furthermore, color‐coordinate readings were acquired against white and black settings to simulate the intraoral light reflectance of natural teeth.^41^ Thus, the differences revealed among the outcomes herein were assumed to be a by‐product of compositional variations among the tested materials (Table 1). Δ*E*_00_ is a well‐established parameter frequently used to quantify the stainability of dental materials subjected to CTC.^22^, ^24^, ^26^ In the present study, significant differences were observed in the stainability among the tested materials after CTC and color correction; while the glass‐ceramics and PICN did not exceed previously reported clinical thresholds,^52^ RNC revealed perceptible color changes after CTC that dissipated upon color correction. Studies have also verified imperceptible color changes of glass‐ceramics after CTC owing to their resistance to hydrolytic degradation by virtue of the densely packed crystalline networks within.^14^, ^15^, ^18^ Some reported superior color stability in ZLSs,^18^ however, others demonstrated higher stain resistance in LDs.^14^, ^19^ Disparities in Δ*E*_00_ findings could be attributed to inconsistencies in Li_2_Si_2_O_5_ crystal size and volume fraction that alter the surface absorption of colorants; EC is comprised of 70 vol% of 1.0–1.5‐µm crystals, whereas VS contains 60 vol% of 0.5–0.7‐µm crystals.^5^, ^6^ Additionally, the ΔE_00_ findings of VE verified its adequate color stability after 1 year of simulated coffee drinking, similar to other studies,^23^ which may be justified by the 86 wt% reinforcement with feldspathic ceramic crystals combined with the decreased monomer content that limited its water uptake. Nonetheless, the Δ*E*_00_ values of VE were greater than those reported by Çakmak et al.^27^ and lower than those reported by others,^14^, ^21^ presumably because of the differences in CTC regimens and polishing protocols. Perceptible color changes in CS herein, align with the findings by Taşın et al.,^14^ and were rationalized by the increased water sorption of bisphenol A‐glycidyl methacrylate monomer present therein, which led to heightened discoloration of CS materials despite the 70% wt Si_2_O filler load. The color correction serum succeeded in reversing the CTC‐induced stains in VS and VE materials, whereas the effect of color correction of EC and Cs was negligible. Previous studies on the same serum have reported short‐term whitening effects; however, prolonged results were not evident.^30^, ^32^ The *L* ^*^, *a* ^*^, and *b* ^*^ varied among the tested materials, notwithstanding their consistent shade and translucency levels; CS were lighter and yellowish, while EC and VS displayed bluish‐green hues.

RTP was quantified based on the CIEDE2000 formula,^18^, ^42^ considering its improved fit of the CIELab color space and the correction of the nonuniformity within. The RTP findings in the present study were contingent on the type of material, whereas the effect of both treatments was irrelevant. Similar RTP values were reported for EC and VS after CTC^18^; however, the RTP values of VE and CS were lower than those stated in the literature,^18^, ^23^ which could be attributed to dissimilar polishing protocols^23^ or different shades. Alp et al.^18^ explored materials in B1 and 1M1 shades, while the present study employed the A1 shade. Therefore, lithium silicate‐based glass‐ceramics may be preferred as a restorative option over PICN and RNC when a more translucent appearance is required. The opalescence of dental materials transpires when the existing refractive index mismatch between the filler and the surrounding matrix exceeds 1.1, which renders visible light scattering and, in turn, the appearance of blue hues upon light reflection and orange hues upon light transmission.^43^ Previous studies reported the range of OP of monolithic CAD‐CAM structures as 5 < OP < 13,^10^, ^17^, ^43^ which was confirmed in the current study. Nonetheless, irrespective of the treatment group, the OP of VE was significantly higher than the other tested materials, which may be credited to the added 10 wt% ZrO_2_ therein. The whiteness index (WI_D_) used in the present study was chosen by virtue of its enhanced ability to objectively quantify whiteness in dental materials.^44^ At baseline, and after color correction, EC and VS exhibited similar WI_D_ values akin to those of esthetic dental materials in other studies,^45^, ^54^ and superior to that of VE and CS herein. When WI_D_ thresholds were considered,^53^ color corrections caused perceptible whiteness differences in EC, VS, and VE, and unacceptable whiteness differences in CS. Nonetheless, these preliminary findings proved that coffee‐induced discoloration of CAD‐CAM materials may be recovered through color correction serums.

GU of all tested materials was significantly reduced after both treatments, which may be a result of the acidic nature of the coffee solution that accelerated the chemical degradation and altered the light reflection and luster of surfaces. Nevertheless, all materials presented acceptable GU values^48^ and minor gloss changes when clinical thresholds were considered (< 20 GU).^47^

The Martens hardness test provides valuable insight into the wear behavior of CAD‐CAM materials through objective computation of their indentation resistance, thereby evading the subjective limitations associated with traditional optical hardness testing methods. In the present study, hardness was measured as a reflection of surface integrity after chemical and thermal exposure of CTC, thereby justifying the concurrent colorimetric changes. The HM findings revealed significantly higher HM among EC and VS compared to VE and CS across all treatment groups, which is consistent with previous findings.^34^, ^39^, ^55^ The disparity in HM among the tested materials is ostensibly conditional on the compositional and filler loading differences therein. Moreover, CTC yielded lower HM in EC and VS, which increased after color correction, possibly because of the manual removal of superficial coffee remnants during tooth brushing.

A main limitation of the current study resides in the nature of CTC, where complete immersion of specimens in the staining solution triggers amplified color changes, whereas the CAD‐CAM restorations are bonded intraorally to underlying substrates and thus are semi‐isolated from staining challenges. Moreover, the use of one thickness, a single staining solution, and no underlying cement could be deemed limitations, as other thicknesses, staining substances, and luting cements may alter the findings.^8^, ^20^, ^56^, ^57^ Another limitation was the concise duration of the color correction treatment; therefore, future studies should explore the impact of prolonged color correction regimes on the outcomes reported herein. Additionally, the specimens were not subjected to glazing or polishing protocols, which could be considered a limitation, as surface treatments may influence the staining resistance and hardness of CAD‐CAM restorative materials. Furthermore, the present findings would have benefitted from an investigation of the concurrent compositional and crystalline changes, using XRD, SEM‐EDX, or Raman analyses. Nonetheless, while a microstructural analysis is valid from a materials standpoint, it goes beyond the scope of this research, which focuses on the assessment of surface properties and clinically perceptible colorimetric changes rather than microscopic compositional alterations per se.

## CONCLUSIONS

Zirconia‐reinforced lithium silicate glass‐ceramics demonstrated superior color stability, opalescence, and hardness when subjected to CTC and color‐correcting serum. Glass‐ceramics displayed greater translucency and whiteness than resin‐based CAD‐CAM restorative materials. The study findings support the promising potential of color correction serums as a temporary solution for the reversal of coffee‐induced staining in CAD‐CAM restorative materials.

## CONFLICT OF INTEREST STATEMENT

The author declares no conflicts of interest.
